# Impact of ATM and SLC22A1 Polymorphisms on Therapeutic Response to Metformin in Iranian Diabetic Patients

**Published:** 2016

**Authors:** Fazlollah Shokri, Hamid Ghaedi, Soudeh Ghafouri Fard, Abolfazl Movafagh, Saeid Abediankenari, Abdolkarim Mahrooz, Zahra Kashi, Mir Davood Omrani

**Affiliations:** 1 *Department of Medical Genetics, Faculty of Medicine, Shahid Beheshti University of Medical Sciences, Tehran, Iran.*; 2 *Immunogenetic Research Center, Faculty of Medicine, Mazandaran University of Medical Sciences, Sari, Iran.*; 3 *Department of Clinical Biochemistry and Genetics, Faculty of Medicine, Mazandaran University of Medical Sciences, Sari, Iran.*; 4 *Diabetes Research Center, Imam Teaching Hospital, Mazandaran University of Medical Sciences, Sari, Iran.*

**Keywords:** Metformin, type 2 diabetes, pharmacogenetic

## Abstract

Metabolic syndrome and its pathological sequel, type 2 diabetes are considered as important global health problems. Metformin is the most common drug prescribed for patients with this disorder. Consequently, understanding the genetic pathways involved in pharmacokinetics and pharmacodynamics of this drug can have a considerable effect on the personalized treatment of type 2 diabetes. In this study, we evaluated the association between rs11212617 polymorphism of *ATM* gene and rs628031 of *SLC22A1* gene with response to treatment in newly diagnosed type 2 diabetes patients. We genotyped rs11212617 and rs628031 polymorphism by PCR based restriction fragment length polymorphism (RFLP) and assessed the role of this polymorphisms on response to treatment in 140 patients who have been recently diagnosed with type 2 diabetes and were under monotherapy with metformin for 6 months. Response to metformin was defined by HbA1c and fasting blood sugar (FBS) values. Based on such evaluations, patients were divided into two groups: responders (n= 63) and non-responders (n= 77). No significant association was found between these polymorphisms and response to treatment (OR= 0.86, [95% CI 0.52–1.41], P= 0.32) for rs11212617 and (OR= 0.45, [95% CI 0.64–1.76], P= 0.45) for rs 628031. The reported gene variants in *ATM* and *SLC22A1* are not significantly associated with metformin treatment response in type 2 diabetic patients in an Iranian population.

Metabolic syndrome and its pathological sequel, type 2 diabetes (T2D) are consid-ered as global major health problems. T2D is a silent, chronic, progressive disease in which both genetic and environmental factors play roles (1, 2). The prevalence of T2D in Asia has increased considerably. International Diabetes Federation data suggest that about 30-60% increase will occur in the prevalence of T2D in many Asian-Pacific countries by the year 2025 (3). In addition, T2D has affected younger population in Asia compared with the Western countries (4). The pathophysiology of T2D is complex. Both β- cell dysfunction and insulin resistance contribute in this disorder with abdominal obesity being a major risk factor for the latter (3, 5). Hyperglycemia, insulin resistance, and hyperinsulinemia have been shown to contribute to increased risk for many malignancies in diabetic patients (6, 7). Accordingly, lifestyle interventions and pharmacotherapy are required to achieve and maintain optimal glucose control and prevent disease related complications (1).

Metformin (1, 1- dimethylbiguanide) is the first- choice and the most widely used drug for treatment of T2D because of its effective, reasonable price and safety (8, 9). Its hypoglycemic mechanisms include reduction of hepatic glucose output, partly via reduced gluconeogenesis, decrease in insulin resistance, especially in liver and skeletal tissue, up-regulation of glucose uptake in adipose tissue, and suppression of the intestinal glucose absorption (3, 6, 10). It also reduces plasma lipid (10). This drug has been used in the treatment of nonalcoholic fatty liver disease, polycystic ovarian syndrome (PCOS), premature puberty as well as prevention of cancer (2-4, 6, 11). It has been shown to influence classical cardiovascular risk factors including LDL-cholesterol, anthropometric indices and blood pressures as well as atherogenic dyslipidaemia, inflammation and vascular function. Furthermore, it improves haemostasis via reduction of factor VII and Factor XIII levels (3) .Additionally, recent studies have indicated an antioxidant effect for metformin (12). Consequently, this drug is an attractive modality for treatment of T2D. However, not all patients benefit from metformin since the hypoglycemic response is not seen in a proportion of patients. Furthermore, gastrointestinal side effects make this drug intolerable in a subset of patients (2). Although variation in response to a certain drug can be attributed to drug–drug interactions, age, organ function, simultaneous therapy, the role of genetic factors in variability in drug effects is significant (13). Metformin has been shown to be actively absorbed from the gut and eliminated unchanged in the urine (2). It is transported into the hepatic cells by organic cation transporter1 (OCT)1 (encoded by *SLC22A1*) (14, 15), and into the renal tubules by OCT2 (encoded by *SLC22A2*) (2, 16). OCT1 has been shown to play a significant role in the efficacy of metformin (4). Population studies have shown a high level of polymorphisms for OCT1 in different ethnicities (2). Functional polymorphisms in the corresponding gene such as rs628031 (Met408Val) have been shown to affect its liver uptake, and consequently influence its efficacy (7, 17). In addition,* ATM* (ataxia telangiectasia mutated) is a gene whose role in DNA repair and cell cycle control is evident. It has also been shown to play a significant role in the modulation of metformin effects, and variations in this gene change the response to this drug (8, 18, 19).

Consequently, in this study we aimed at analysis the association between rs11212617 polymorphism of *ATM* and rs628031 of *SLC22A1* genes and glycemic response to metformin in an Iranian population of diabetic patients.

## Materials and methods

**Patients**

This study included 140 patients (121 women and 19 men)with newly diagnosed T2D according to WHO (20). The mean age of the patients was 53.06 (±18) years. Clinical characteristics of patients, including weight, height, blood pressure, and BMI, are presented in Table 1. All patients received metformin (1000mg/day) for a 6 month period. None of the patients were receiving insulin therapy or oral anti-diabetic (ODA) medication prior to their diabetes diagnosis. Exclusion criteria for the study were type 1 diabetes, chronic hepatic disease, renal failure, autoimmune diseases, malignant diseases, and pregnancy. The protocol study was performed in accordance with the ethical standards of the institutional ethics committee and with the Helsinki Declaration of 1975, as revised in 2008. Informed consent was obtained from all participants before enrollment. All patients underwent a physical examination, and information about medical history, demographic parameters, and medication use was obtained using a questionnaire. Most patients had a family history of diabetes, and taking antihypertensive medication such as losartan, an ACE inhibitor, or a beta blocker, and most patients were receiving lipid-lowering therapy.

Based on the response to metformin, patients were classified into two groups: responder group (who showed a decrease in HbA1c levels by at least1% from the baseline) and non- responder group.

**Laboratory analyses**

The HbA1c levels were assayed by boronate affinity technique (Axis Shield PoC AS, Oslo, Norway; accuracy, failure<5 %). Standard enzym-atic tests were used to assay values of fasting blood sugar (FBS), triglycerides (TGs), total cholesterol (TC), HDL-C, ALT, and AST after an overnight fast. The LDL-C values were calculated according to the Friedewald method (21).

**Genotype determination**

Genomic DNA was extracted from samples containing EDTA. PCR-based restriction fragment length polymorphism (RFLP) was used to genotype the mentioned variant. In brief, DNA was amplified in 25 µL of reaction mixture consisted of 1 unit of Taq DNA polymerase, 400- 500 ng genomic DNA, 200 mM of each dNTP, 1.5 mM MgCl2, 280 nM of each primer. The designed primers used for *SLC22A1*- rs628031 polymorphism were F5'- CTAAACCCAGTGATTCATGCTCTTT- 3' and R5^'_^ TTTGTTCTCATTCCAGAGGCTTATC -3^'^, and for *ATM*- *rs11212617 *polymorphism were F5’^_ ^TGGGTTGCTTGTGGATAACATATAGTTGG- 3^'^and R5^'^- GAGAAGGCAGTAAAGTGAAGG-AATACAGAG- 3^'^.

PCR for *SLC22A1*- rs628031 polymorphism was accomplished at 95 °C for 5 min, followed by 35 cycles of 95 °C for 30 s, 64 °C for 35 s, and 72 °C for 60 s, with a final extension step of 72 °C for 5 min. Amplification products from each sample (422 bp) were cleaved by MscI (Fermentas, Lithuania) after 15 h incubation at 37 °C and resulted in 154 and 268- bp fragments, which were subjected to electrophoresis on a 2% agarose gel.

PCR for *ATM*- rs11212617 polymorphism was accomplished at 95 °C for 5 min, followed by 35 cycles of 95 °C for 30 s, 64.5 °C for 35 s, and 72 °C for 60 s, with a final extension step of 72 °C for 5 min. Amplification products from each sample (209 bp) were cleaved by TaaI (HpyCH4III) (Fermentas, Lithuania) after 15 h incubation at 65 °C and resulted in 153 and 56- bp fragments, which were subjected to electrophoresis on a 2 % agarose gel.

**Statistical analyses**

All statistical analyses were performed by statistical software package for social sciences (SPSS 18.0, Chicago). The clinical and laboratory data were expressed as mean± SD or percentages. To determine variable distributions, we used Kolmogorov Simonov normality test. Mann–Whitney U test was used to analyze differences of non- parametric variables. The association between categorical variables, such as genotype groups and metformin response was determined with chi- square test. The odds ratios (ORs) and 95% confidence intervals (CIs) were calculated as a measure of the association of *SLC22A1*-rs628031and *ATM*- rs11212617 variants with metformin response. The chi-square goodness-of-fit test with one degree of freedom was used for testing Hardy–Weinberg equilibrium. P values ≤ 0.05 were considered to be statistically significant.

## Results

In this monotherapy study, the subjects were split into two groups: responders (n= 63) and non-responders (n =77). The groups did not differ significantly in age (53.68± 9.68 years in the responder group, 52.96± 10.34 years in the non-responder group, P= 0.51). Of the 140 participants, 121 were women (55 were responders and 66 were non- responders) and 19 were men (8 were responders and 11 were non- responders). Values of the study parameters at baseline and after metformin therapy based on responder and non- responder status are presented in Table 1.

As shown in Table 1, there was a statistically significant difference between responders and non-responders after metformin therapy with respect to systolic blood pressure (SBP) and diastolic blood pressure (DBP). The allele frequencies and genotypes distribution of *ATM*- rs11212617 and *SLC22A1*- rs628031 polymorphisms are shown in Table 2 and 3, respectively.

**Table 1:** Values of the study parameters at baseline and after metformin therapy based on responder and non- responder status

**After 25 Weeks**			**Baseline**			
**P-value**	**Responders**	**Non-Responders**	**P-value**	**Responders**	**Non-Responders**	**parameter**
0.51	53.68± 9.68	52.96±10.34	0.51	53.68± 9.68	52.96±10.34	Age
0.04	122.38±22.46	127.82±18.34	0.13	129.49±16.12	135.02±15.03	SBP (mmHg)
0.03	76.44±10.34	79.65±10.16	0.20	79.32±10.27	90.96±79.67	DBP (mmHg)
0.54	74.90±14.02	78.37±15.81	0.73	76.23±13.88	79.28±15.85	Weight
0.39	1.57±0.07	1.58±0.08	0.39	1.57±0.07	1.58±0.08	Height
0.88	30.43±5.70	31.12±5.49	0.84	30.94±5.62	31.49±5.58	BMI
0.00	118.91±19.33	143.22±40.33	0.68	146.72±28.54	142.10±22.77	FBS (mg/dL)
0.00	6.29±0.70	7.64±1.15	0.03	7.96±0.80	7.54±0.81	HbA1C (%)
0.36	152.55±55.39	174.34±88.84	0.29	185.38±82.64	171.60±81.14	TG1 (mg/dL)
0.75	167.64±33.75	176.70±35.29	0.58	185.86±38.99	182.55±43.70	TC (mg/dL)
0.77	49.83±16.28	48.14±13.02	0.11	46.03±15.00	48.83±15.03	HDL (mg/dL)
0.98	86.64±26.93	90.74±23.39	0.60	101±34.36	99.39±34.73	LDL (mg/dL)

SBP: systolic blood pressure; DBP: diastolic blood pressure; BMI: body mass index; FBS: fasting blood sugar; TG1: triglyceride fraction 1; TC: total cholesterol; HDL: high-density lipoprotein; LDL: low-density lipoprotein

**Table 2:** Genotypes and alleles frequencies of *ATM*- rs11212617

	***Responder n (%)***	***Non-Responder n (%)***	***OR (95% CI)***	***P***
*Genotype *				
TT	23 (36.50)	36 (46.75)	0.65^a^ (0.33-1.29)	0.14
TG	34 (53.97)	31 (40.26)	1.74^b^ (0.88-3.41)	0.07
GG	6 (9.53)	10 (12.91)	0.61^c^ (0.20-1.85)	0.27
*Allele *				
T	80 (63.49)	103 (66.88)		
G	46 (36.51)	51 (33.12)	0.86^d^ (0.52-1.41)	0.32

a AA versus Aa+aa,

b Aa versus AA+aa,

c aa versus AA+Aa,

d A versus a

**Table 3:** Genotypes and alleles frequencies of *SLC22A1*- rs628031

	***Responder n (%)***	***Non- Responder n (%)***	***OR (95% CI)***	***P***
*Genotype *				
AA	29 (46.03)	37 (48.05)	0.92^a^ (0.47-1.79)	0.47
AG	28 (44.44)	29 (37.66)	1.32^b^ (0.67- 2.60)	0.26
GG	6 (9.52)	11 (14.28)	0.63^c^ (0.22-1.81)	0.27
*Allele *				
A	86 (68.25)	103 (66.88)		
G	40 (31.75)	51 (33.12)	1.06^d^ (0.64-1.76)	0.45

a AA versus Aa+aa,

b Aa versus AA+aa,

c aa versus AA+Aa,

d A versus a

## Discussion

In this study, we have defined the response to metformin in 140 patients with T2D. Contrary to our hypothesis, no association was found between variants in *SLC22A1* and *ATM* genes, and glycemic response to metformin.

There is a huge clinical variation in response to metformin, and this drug is usually combined with other agents such as sulfonylureas to treat diabetes. Clinical trials data have indicated that more than one third of patients receiving metformin monotherapy do not achieve acceptable control of fasting glucose levels (22). The main reason for the lack of a dramatic response in the treatment of these patients may be a variation of genes involved in pharmacokinetics and pharmacodynamics of the drug (2, 23, 24).

It has been demonstrated that the OCTs and ATM proteins play a dominant role in glycemic response to metformin (14, 15, 18).

HbA1c level has been assessed as a marker of treatment response in diabetic patients in many studies. In some of them, treatment success has been defined as the ability to reach the treatment target of an HbA1c≤ 7 % (8, 18, 19). However, Shikata et al. selected reduction of HbA1c values by more than 0.5 % as a cut off point for dividing patients into responders and non- responders (17). As reported in a systematic review, over a 3-month period of metformin therapy, HbA1c values decreased by approximately 1% compared with placebo. Data of previous studies of OAD drugs indicate that they reduce HbA1c levels by 0.5–1.5% (1). Thus, in the present study, a reduction of 1≤ % in HbA1c after 25 weeks was deemed a response to metformin therapy.

In our study, the effect of metformin in the treatment of diabetes or improvement of relevant glycemic traits was not magnified among the carriers of the C allele at rs11212617 in the *ATM* gene. Our finding concurred with a previous report which showed no association between this polymorphism and insulin sensitivity, fasting glucose, HbA1c, or disposition index diabetes prevention program (DPP) (8). However, this finding does not support the previously reported association of this allele with improved metformin action on glycemic control (18). A previous study conducted in T2D patients in the Netherlands and the UK has identified rs11212617 as the first robustly replicated common susceptibility locus associated with metformin treatment response (19). However, the function of this gene variant is not elucidated so far (25).

Although OCT1 encoded by *SLC22A1* gene has been shown to play a significant role in the efficacy of metformin, the association of rs628031 variant with glycemic response has not been assessed before. So, our study is the first study in this regard which shows no significant association between rs628031 alleles and metformin response. In the present study, the frequency of the mutant allele of *SLC22A1*-rs628031(M408V) variant between responders and non-responders was not significantly different. The M408V variant has been associated with gastrointestinal side effects in 246 metformin users. A significant lower average HbA1c level in the presence of a lower average dose of metformin in those cases implies a possible relationship between better response to metformin and susceptibility to side effects (26). The local increase of drug concentration in the intestinal tissue is proposed as a mechanism of metformin intolerance. As *SLC22A1* and *SLC22A3* are also expressed in enterocytes, it has been suggested that genetic variants in these genes may also affect the intestinal metformin uptake and consequently induce gastrointestinal side effects (14).

Previous studies showed that OAD drugs effectively decrease HbA1c levels by 0.5–1.5 % (1). This result demonstrates that the greater proportion of the decrease in HbA1c levels in all patients after metformin therapy was associated with responders, suggesting that metformin response can be important in evaluating HbA1c as a key indicator in monitoring the long- term glycemic control.

The limitation of our present study was that we did not assay plasma metformin concentrations to examine the link between the metformin levels and the allelic and genotypic distribution of the studied variant.

Inconsistent results in the mentioned studies could be due to multiple reasons including the different definitions of metformin responses in different studies, the different efficacies for metformin at different HbA1c baselines, and more importantly variations in different populations.

In summary, future large- scale studies are needed for the identification of novel loci affecting treatment response especially considering the fact that the biology of metformin working mechanism

 is not fully understood.

In addition, a more complete investigation of *SLC22A1* variants may identify novel polymorphisms which can affect the metformin response. Additional investigations would identify the ethnic variability in the* SLC22A1* and *ATM* genes and the inter- individual differences in response to metformin.
